# Removal of Heavy Metals from Acid Mine Drainage by Red Mud–Based Geopolymer Pervious Concrete: Batch and Long–Term Column Studies

**DOI:** 10.3390/polym14245355

**Published:** 2022-12-07

**Authors:** Wenbin Xu, Hailang Yang, Qiming Mao, Lin Luo, Ying Deng

**Affiliations:** 1 College of Water Resources and Civil Engineering, Hunan Agricultural University, Changsha 410128, China; 2 College of Resources and Environment, Hunan Agricultural University, Changsha 410128, China

**Keywords:** acid mine drainage, geopolymer pervious concrete, red mud, heavy metals

## Abstract

Various metal ions in acid mine drainage (AMD) cause environmental pollution. Due to the unique advantages of heavy metal treatment and gelling properties, previous concretes incorporating red mud have attracted extensive attention in AMD passive treatment, which utilises naturally occurring chemicals to cleanse contaminated mine waters with low operating costs. This study aims to develop red mud–based geopolymer pervious concrete as an eco–friendly method to remove heavy metals in AMD. Compared with raw pervious concrete, red mud–based geopolymer pervious concrete improves the purification efficiency of heavy metals. The high rate of acid reduction and metal removal by the geopolymer is attributed to the dissolution of portlandite in red mud. Precipitation of metal hydroxides seems to be the dominant metal removal mechanism. Under optimal conditions (influent pH = 4.0 and the hydraulic retention time = 24 h), red mud–based geopolymer pervious concrete could completely remove Cu(II), Mn(II), Cd(II) and Zn(II) by up to 10 mg/L, 10 mg/L, 1.6 mg/L and 16 mg/L, respectively. When the influent pH is 2.5, the hydrolysis of Fe(III) released from red mud increases the consumption of OH^−^. Moreover, when the influent pH is 4.0, the precipitation of CaSO_4_ promotes the dissolution of portlandite and metal removal. Therefore, red mud has demonstrated feasibility in the manufacturing of geopolymer–based pervious concrete for purification AMD.

## 1. Introduction

Mining activities in China contribute significantly to the economy, among which nonferrous metals are an important component [1]. In Hunan Province, large–scale mining for non–ferrous metals takes place, consequently followed by a large number of acid mine drainage (AMD) treatments. In particular, the non–point source pollution of AMD produced by mining after rain is a problem that needs to be solved urgently. The main characteristics of AMD are a low pH value, excessive concentration of heavy metals, variation in the type of heavy metals, unstable water flow and a large number of sulphate ions [2]. For example, at Qibao Mountain, Liuyang City, Hunan Province, the pH is approximately 4–6, the concentration of sulphate ions is up to 800 mg/L and there is an excessive presence of heavy metals including Cd, Mn, Cu and Zn. AMD can easily enter rivers and lakes through production and confluence of runoff, polluting agricultural irrigation and drinking water, and also causing damage to natural ecology and human health [2]. Chief treatment measures at present are focused on passive ecological interception, which uses an interception ditch system to collect AMD and achieves its treatment through stabilisation, interception and advanced treatment. This includes the chemical curing effect by enriched plants and chemicals present in the soil [3]; interception of ecological ditches and ponds [4]; and biological adsorption through enhancement of ecological filters [5]. While these systems have great advantages, such as low cost and simple maintenance, there are significant problems, such as poor effectivity during winter and unstable water output. Therefore, passive treatment materials which are effective and low cost should be developed to ensure the stable treatment of AMD.

Pervious concrete is a commonly used material in hydraulic engineering, and exhibits a good interception effect on water pollution owing to its honeycomb void structure, making it a prospective material for sewage treatment [6,7,8]. Pervious concrete has a significant effect on nitrogen and phosphorus interception in water and is widely used in ecological slope protection projects [9]. In recent years, several studies have explored the interception effect of pervious concrete on AMD. Haselbach et al. and Kaur et al. conducted long–term experiments to simulate its potential for treating Cu and Zn (Zn: 0.1 mg/L and Cu: 0.02 mg/L) and Zn, Fe and Mn in rain and underground water (flooding velocity was 0.35 mL/min), respectively [10,11]. Calkins et al. conducted a 24 h heavy metal treatment test and found that the removal rate of Cu from the solution by pervious concrete could reach 50–60%, extending the application range of pervious concrete; however, further research on its sustainability was lacking [12]. Holmes et al. studied the influence of aggregate, cement and fly ash on the removal of heavy metals, and after 72 h of testing, they found that using fly ash with excessive sulphur trioxide as an admixture to prepare pervious concrete improved the ability of pervious concrete to remove Pd, Cd and Zn from the solution [13]. Wu et al. enhanced the ability of pervious concrete to remove heavy metals by adding diatomaceous earth, which caused the cementitious material in pervious concrete to have a more significant impact on the interception effect on heavy metals than on aggregates [14]. However, the various types of pervious concrete studied have limited adsorption rates for heavy metals, and their application effect and conditions in non–point source pollution from mining have not been explored sufficiently; thus, a modification focusing on replacing cementitious material with alternatives needs further exploration.

Red mud is a type of aluminium waste with a high output [15]. About 120 million tons of red mud were produced annually worldwide, 90% of which came from China and Australia [16]. Governments and enterprises have been facing problems regarding storage and management of red mud. In recent years, researchers have explored its potential use as a cementitious binder in concrete mixes [15]. Chen et al. found that red mud can be successfully used as a part of aluminosilicate (less than 50%) to form geopolymers in concrete, resulting in a new red mud–based geopolymer pervious concrete (RMPC) [17]. Saravanan et al. summarised the research on the replacement of red mud in cementitious concrete, and a 10–15% replacement of red mud and 5% of hydrated lime with cement showed good results [18]. Intriguingly, recent research has shown that red mud is effective for treating heavy metals in sewage, owing to its large surface area (between 11.65 and 30.72 m^2^/g) and strong basicity (pH between 9.2 and 12.8) [17]. Therefore, low–cost, green and sustainable treatment materials are a hot topic of research [19], which can be obtained by using the characteristics of red mud to modify pervious concrete. However, at present, only a few studies have focused on this material for heavy–metal interception. Therefore, it is crucial to systematically study the effectiveness and applicability of RMPC for the control of heavy metal non–point source pollution from mining and propose methods for its application.

This study aimed to use red mud to replace cement and form RMPC. The heavy metal interception ability and applicable conditions of RMPC were determined through various testing systems to explore its application in heavy metal non–point source pollution from mining. Its effectiveness was verified through monitoring of its actual application for approximately 32 weeks. The mechanism of heavy metal removal by RMPC was analysed by Fourier transform infrared spectroscopy (FT–IR), X–ray diffraction (XRD) and scanning electron microscopy (SEM). As a result, this article provides suggestions on the application of RMPC in practical projects. This provides a novel alternative technology for recycling of red mud, as well as a low–cost, stable and efficient treatment material for heavy metal non–point source pollution from mining.

## 2. Materials and Methods

### 2.1. Preparation of Materials and Samples

In this study, coarse aggregate, cement, red mud, water and other materials were used to prepare pervious concrete. The associated oxide compositions of Portland cement (OPC) and red mud (RM) were determined using X–ray fluorescence (XRF), as shown in Table 1. The cement used was ordinary Portland cement, and the red mud was the product obtained from the Zhuzhou aluminium–alumina process, using polycarboxylic acid polymer powder as a water–reducing agent.

To make RMPC acquire appropriate permeability, its porosity was controlled at 20–30% [20], and the water–to–cement ratio and admixture dosage were determined through multiple tests. The specific mix ratio designs are listed in Table 2. The mould size used for tests was 100 mm Ã–100 mm Ã–100 mm. To obtain standard samples, pervious concrete was prepared by hand mixing, demoulded after 24 h of film coating, and placed in a constant temperature curing room at 20 ± 2 °C until curing was completed (after 28 days). Eighteen cubes of each mixture were prepared.

### 2.2. Strength, Porosity and Permeability Tests

To ensure that the prepared pervious concrete materials could meet the engineering application standards, the 28–day compressive strength test of 100 mm × 100 mm × 100 mm cubes was conducted according to “Standard for test method of mechanical properties on ordinary concrete (GB/T50081-2002)”. The permeability coefficient was determined according to “Permeable paving bricks & permeable paving flags (GB/T25993-2010)”. The porosity was determined according to the gravimetric method. Three repeated experiments were performed for each group.

### 2.3. Heavy Metal Removal Experiment

**Figure 1:**
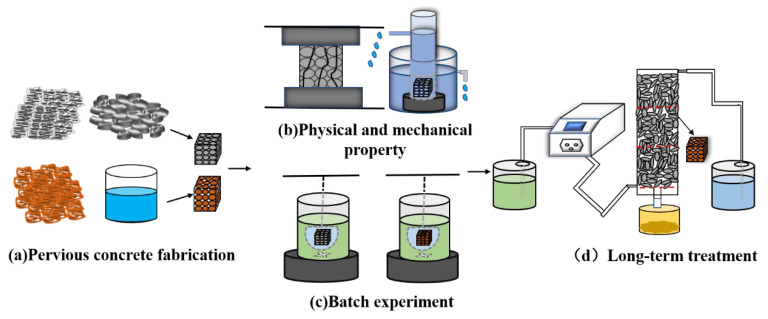
Experimental flow diagram.

To explore the effects of RMPC and raw pervious concrete on heavy metals in solutions corresponding to concentrations present in the heavy metal pollutes at Qibaoshan, the relevant masses of MnSO_4_•H_2_O, ZnSO_4_•7H_2_O, CdCl_2_•2.5H_2_O and CuSO_4_•H_2_O were dissolved in ultrapure water to prepare simulated acid mine drainage. The initial pH values and elemental concentrations are listed in Table S1. Referring to the experimental process by Holmes et al. (Figure 1) [13], batch experiments for heavy metal removal were carried out using a magnetic stirrer (rotation speed of 500 rpm), and samples were collected every 15 min until the end of the experiment. Immediately after collecting the treated samples, the pH of the wastewater was measured. Next, ICP–OES was used to determine the equilibrium concentrations of the individual metals. Each group of concrete cubes (including the control group) were tested in triplicate.

RMPC was used for the long–term dynamic column test. Referring to Shabalala’s method [6], peristaltic pumps were used to regulate the water flow velocity and Perspex columns were used to simulate the actual inlet and outlet water environments (Figure 1). A water inlet was present on the side of each Perspex column and an outlet was present along its height, which were used for controlling the input and output of the solution, respectively. Three concrete cubes were placed in each column and the solution was pumped from the storage bottle to the Perspex column using a peristaltic pump. During the test, pH, heavy metal concentration, hydraulic retention time and other conditions of the input solution were regulated to determine the influencing factors and optimal conditions for heavy metal removal. The samples were collected every day, and the heavy metal detection procedure was the same as described above.

### 2.4. Mineralogical and Microstructure Characterization

The microscopic and material property tests of the concrete changing process in the long–term column tests were performed as follows.

Precipitate samples were collected every month (30 days per cycle) for drying treatment. XRD (XRD–6100, Shimadzu, Kyoto, Japan) was measured at a measurement range of 10–80°, step size of 0.03° (2 θ) and scanning step size of 0.6 s. Powder samples (5 g) were taken for scanning. Jade software was used to analyse the XRD spectrum of the samples, and Origin was used for plotting.

Concrete samples before and after the reaction and precipitate samples collected each month were analysed with FT–IR (Nicolet–iS5, Nigaoli, Madison, WI, USA) with a scanning range of 500~4000 cm^−1^ and spectral resolution of 4 cm^−1^. Linear baseline correction of the spectrum was performed automatically. Light transmittance was converted into absorbance to obtain the FT–IR absorption characteristic spectra of concrete and precipitate samples and plotted using Origin 8.0 (OriginLab, Northampton, MA, USA).

SEM (Regulus8100, Hitachi, Tokyo, Japan) and energy dispersive X–ray spectrometry (EDS, Octane Elect Super C5, EDAX, Pleasanton, CA, USA) were used on cross–sections of the concrete cubes for heavy metal analysis to study the changes in its microstructure and elemental composition, and qualitatively estimate the physical and chemical processes of heavy metal removal by concrete. This process also provided the microscopic morphology for validating the test results of XRD and FT–IR.

### 2.5. Practical Engineering Application

The mine leaching wastewater from Hunan Province was collected, and a column experiment device was used to conduct interception tests of wastewater for 15 days, to observe the treatment effect of heavy metal removal under actual conditions, and evaluate the cost of RMPC use to determine its value for application in practical engineering.

## 3. Results and Discussion

### 3.1. Mechanical Properties of RMPC

**Figure 2:**
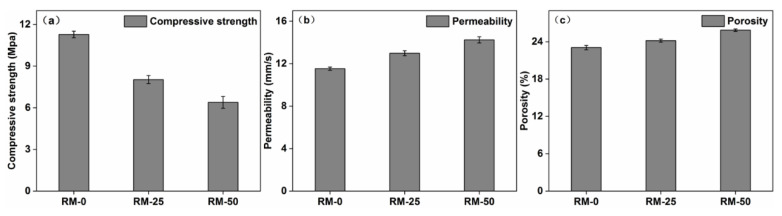
(**a**) Compressive strength, (**b**) permeability and (**c**) porosity of RMPC with different red mud contents.

As the red mud content increased from 0 to 50%, the compressive strength of RMPC decreased by approximately 40%. Figure 2a shows the 28–day compressive strength of the RMPC. Red mud is a highly alkaline solid waste; its Na_2_O content being higher than that of cement (Table 1), which may lead to less C–S–H (Ca_5_Si_6_O_16_(OH)·4H_2_O) being produced during hydration resulting in the reduced strength of the binder. However, because a large number of reactions occurred in the early stage, the rate of strength growth dropped sharply in the later stage. The final compressive strength of concrete was much lower than that obtained in the experiments of Senff et al. [21]. Particularly, when the content of red mud was over 50%, the pervious concrete did not meet the requirements for engineering applications. Thus, pervious concrete with 0–50% red mud content is optimal for such applications.

The porosity and permeability increased slightly with an increase in the red mud content, which facilitated application of RMPC. Figure 2b,c shows the permeability coefficient and porosity of RMPC. The highly alkaline oxide abundant in red mud reacted quickly with water at the beginning. Concrete mixtures were dry and uneven during mixing, which resulted in a larger local pore structure. The permeability also improved because of this reason. The specific surface area of red mud is much higher than that of ordinary cement. Many loose and small pore structures were produced on its outer surface, which also improved the porosity and permeability coefficient. The above results showed that RMPC (with 0–50% red mud content) can be applied in general engineering.

### 3.2. Heavy Metal Removal Characteristics

#### 3.2.1. Effect of Red Mud Content on Heavy Metal Removal by Geopolymer Pervious Concrete

**Figure 3:**
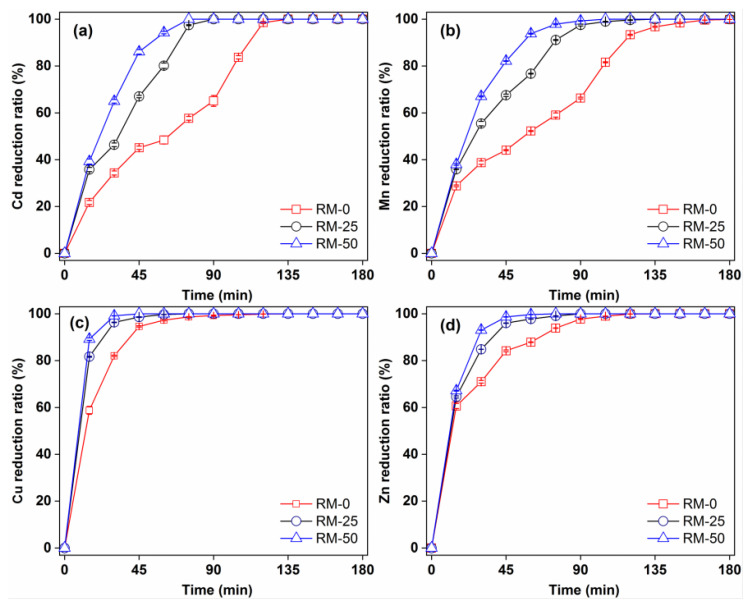
Time–dependent removal of (**a**) Cd, (**b**) Mn, (**c**) Cu and (**d**) Zn in RMPC with different red mud contents.

Figure 3 shows the removal efficiency over time for Cd(II), Mn(II), Cu(II) and Zn(II) by RM-0, RM-25 and RM-50. The removal of these metals depended on reaction time and type of concrete (raw pervious concrete or RMPC) used. The Cd(II) and Mn(II) removal efficiencies were at a maximum (approximately 100%) after 60 min application of RM-25 and RM-50, while it was reached after 135 min with RM-0. The Cu(II) and Zn(II) removal efficiencies tended towards the maximum after 45 min application of RM-25 and RM-50, while it reached a maximum after 90 min with RM-0. This indicated that the addition of red mud improved the removal rate of heavy metals by pervious concrete. Studies have shown that increasing red mud content in geopolymer pervious concrete improves the adsorption of heavy metal ions significantly [17]. Red mud is composed of fine particles with high surface reactivity, which improves the adsorption ability of pervious concrete for heavy metals [22,23]. In addition, the lattice structure of haematite in red mud can trap heavy metals [24]. Once they are fixed within the lattice structure of hematite, the heavy metals are no longer water–soluble or exchangeable [24]. The alumina in red mud participates in the precipitation reaction of heavy metals, and its high alkalinity neutralises the acid in the system, creating conditions for ion exchange [24]. In addition, geopolymeric gel and C–S–H gel with a lower Ca/Si ratio also contributes to the heavy metal removal performance of RMPC [17].

#### 3.2.2. Heavy Metal Treatment Efficiency of RMPC under Various Conditions

**Figure 4:**
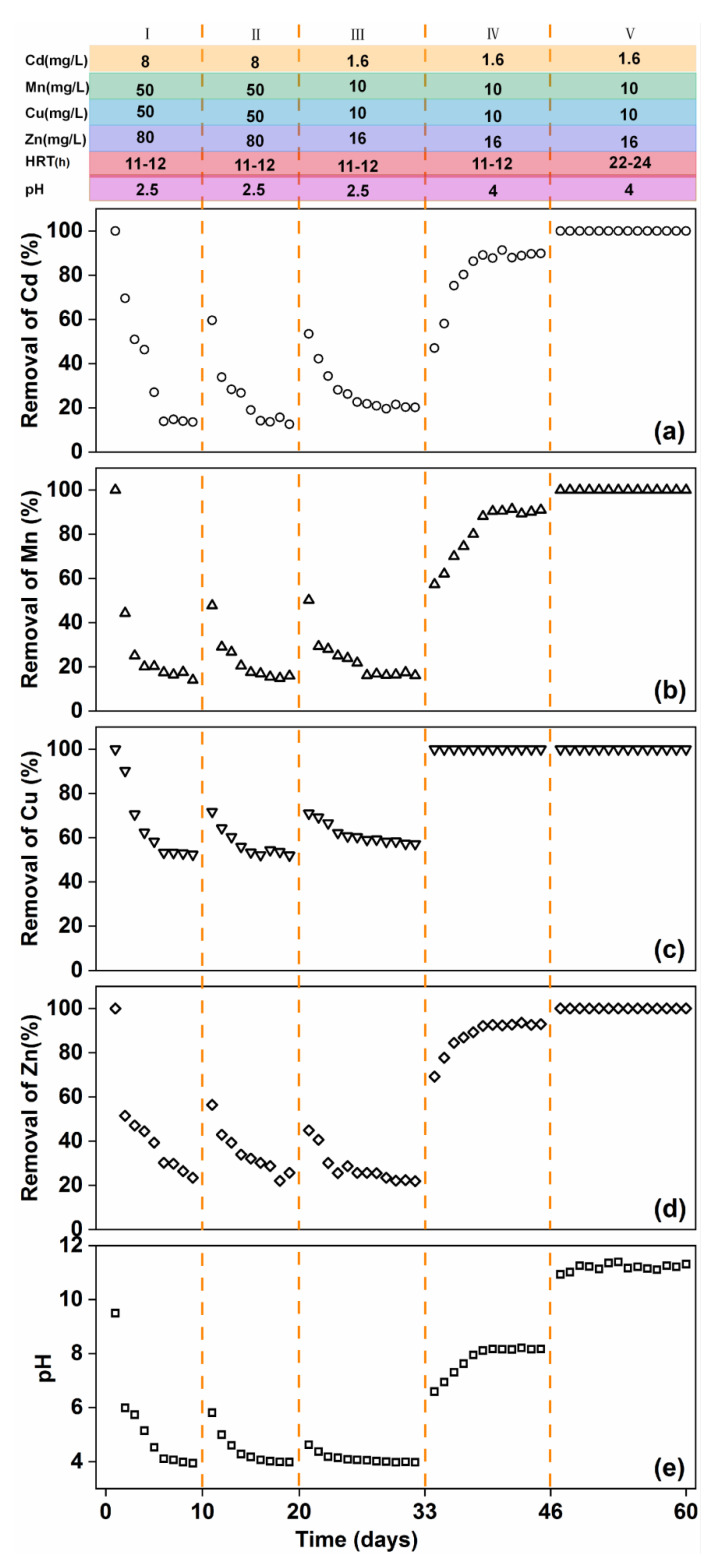
Time–dependent removal of (**a**) Cd, (**b**) Mn, (**c**) Cu and (**d**) Zn, and (**e**) pH from simulated acid mine drainage by RMCP under different conditions.

Influent conditions markedly influenced the treatment of heavy metals by RMPC. Four different conditions were screened at four stages of the test, and the influence of these conditions was analysed, as shown in Figure 4. In Stage I, the effluent, after passing through the pervious concrete, achieved complete removal of heavy metals. With an increase in the continuous working time of the RMPC, the removal efficiency of heavy metals decreased. The removal efficiency decreased to less than 20% after a certain amount of time. Heavy metal removal efficiency was insufficient under such conditions. Thus, technical adjustments are required to ensure long–term application of RMPC. In Stage II, the experiment was repeated after precipitate removal. Results show that the removal efficiency increased briefly and then decreased rapidly. Therefore, while precipitate is produced during the interception process, it is not the key factor affecting removal efficiency. In Stage III, the effluent concentration was reduced. Removal efficiency of heavy metals was almost the same as that in Stage I. Even when RMPC was applied for some time, the removal efficiency decreased. The initial concentration of heavy metals in the influent had little influence on the removal of heavy metals. The above three stages of tests showed a common feature, that is, the pH of the effluent discharged from the outlet gradually decreased with the increase in service time and finally reached approximately 4.0. The trend of heavy metal removal efficiency in the effluent was consistent with that of the pH. Therefore, the influent pH was constantly adjusted in the experiment. In Stage IV, it was found that when the pH of the effluent exceeded 4.0, the removal efficiency of heavy metals was completely different. With an increase in the RMPC service time, the pH of the effluent discharged from the outlet and the removal efficiency of heavy metals gradually increased until the pH was constant at approximately 8.0. The removal efficiency of each heavy metal stabilised at above 90%. Therefore, the RMPC may continuously achieve complete removal of heavy metals in weakly acidic influent (pH ≥ 4.0). The mechanism is strongly associated with pH, which supports the heavy metal removal efficiency of RMPC at pH ≥ 4.0.

In Stage V, to further ensure the stability and complete removal of heavy metals, the hydraulic retention time was increased. Results showed that the pH of the discharged effluent remained stable for 15 days and the removal of Cd(II), Mn(II), Cu(II) and Zn(II) was almost complete. Thus, the concentrations of heavy metals in the discharged effluent can achieve the national discharge standard, and RMPC successfully performed the function of heavy metal treatment under these conditions.

#### 3.2.3. Long–Term Effectiveness of RMPC

Based on the above results, RM-50 was placed in the cylinder to determine its application in engineering and its long–term effectiveness. The results in Figure S2 show that RMPC exhibited good removal efficiency for the four heavy metals during the 228 days (seven months) of the test. With an increase in service time, the removal efficiency fluctuated slightly. At 228 days, the concentrations of Cd, Mn and Zn in the effluent were 0.1mg/L, 0.4 mg/L and 0.1 mg/L, respectively, and the Cu content was almost 0. According to the national standard “Integrated wastewater discharge standard (GB 8978-1996)”, the effluent concentration met the discharge standard. At 228 days, RM-50 had undergone 32 weeks of simulation testing. Its treatment efficiency increased by 3–5 times when compared to the traditional PRB device [6,25], proving that it could achieve better engineering applications under these conditions.

### 3.3. Mechanisms of Heavy Metal Removal by RMPC

#### 3.3.1. Characteristics of Precipitates

**Figure 5:**
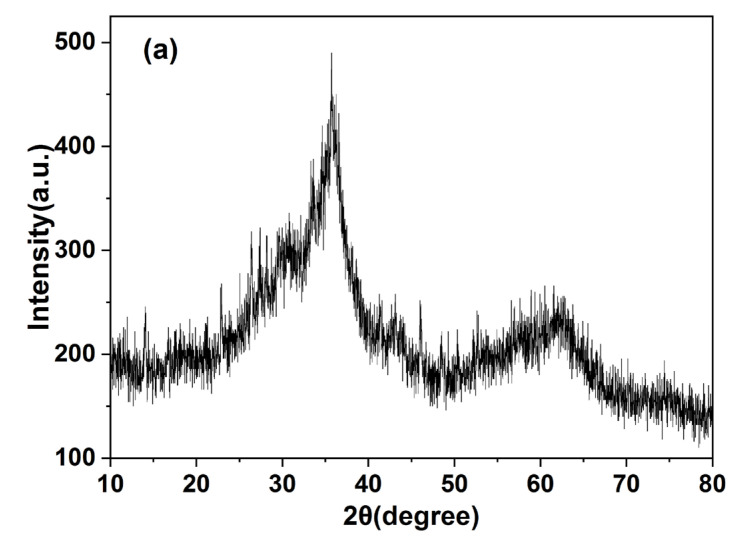

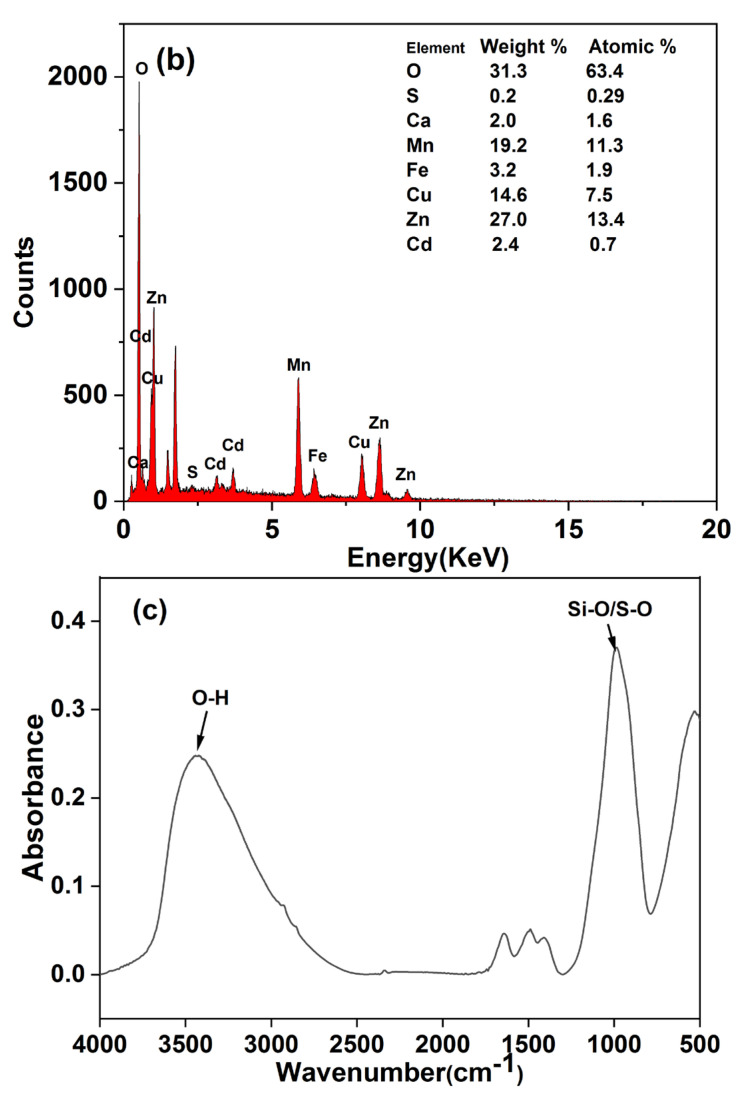
(**a**) XRD, (**b**) EDS and (**c**) FT−IR patterns of precipitates.

The XRD, EDS and FT–IR spectra of the precipitates are shown in Figure 5. EDS spectra showed that the precipitate was mainly composed of O, Mn, Zn, Cu, Cd and other elements, indicating that heavy metals had been precipitated out. Ca, S and Fe were also observed. The precipitate produced was characterised using an X–ray diffractometer (Figure 5a), and XRD images consistent with those of ferrihydrite were obtained [26]. A large amount of Fe(III) in the red mud was hydrolysed and formed one of the precipitates. Figure 5c shows the Fourier spectrum of precipitate, where absorption peaks were concentrated near 3420 cm^−1^ and 990 cm^−1^, and three weak absorption peaks appeared at 1318–1635 cm^−1^. The strong absorption peak at 3420–3440 cm^−1^ was caused by the O–H stretching vibration [27,28], and the peak near 1620 cm^−1^ was caused by the O–H bending vibration [29,30]. Therefore, it was proven that a large amount of hydroxide was present in the precipitate. The peak near 1400 cm^−1^ was caused by the C–OH bending vibration [31,32], which is present due to formation of a polymeric precipitate with iron. The absorption peak at 988 cm^−1^ was caused by the stretching vibration of Si–O and S–O [31], which are silicate and sulphate precipitates. It was inferred that that calcium silicate and calcium sulphate were precipitated.

Thus, the following changes were inferred for the process of wastewater flowing through the pervious concrete: Fe(III) in the red mud hydrolysed to form ferrihydrite precipitate; the heavy metals in the solution reacted with alkaline substances to form precipitates such as Mn(OH)_2_, Zn(OH)_2_, Cu(OH)_2_ and Cd(OH)_2_; and the SO_4_^2−^ in the solution and precipitated Ca(II) formed CaSO_4_ precipitate. One of the main removal mechanisms of heavy metals by RMPC was the formation of hydroxide precipitate by a chemical reaction.

#### 3.3.2. Material Composition and Microstructure of RMPC

**Figure 6:**
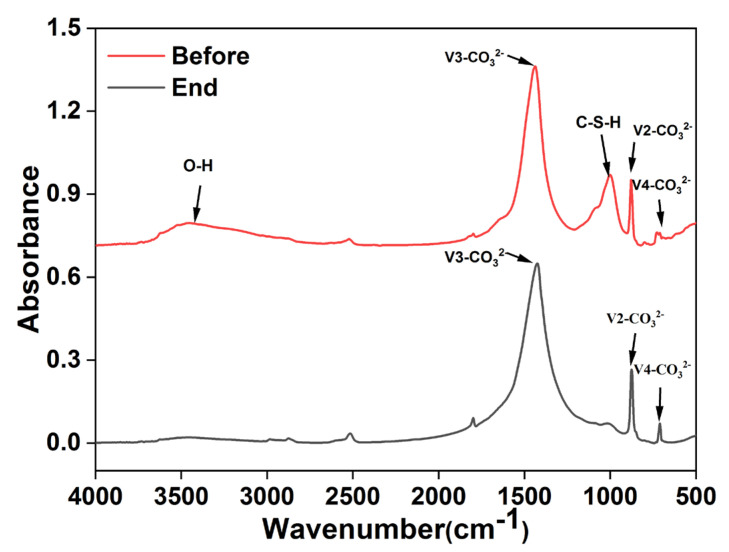
FT−IR pattern of RMPC before and after reaction.

The FT–IR image of RMPC was obtained by superimposing the spectral curves of the various substances, as shown in Figure 6. The sharp absorption band near 875 cm^−1^ corresponds to the bending vibration of V2–CO3^2−^, the band in the range of 1400–1450 cm^−1^ corresponds to the stretching vibration of V3–CO3^2−^, and the band near 720 cm^−1^ is the bending vibration of V4–CO3^2−^ [27,33]. The main component was carbonate in the aggregate, which showed no significant change before and after the test. It was confirmed that the aggregate did not react with heavy metals during the test. The absorption band at 1001 cm^−1^ observed before the test was attributed to the presence of C–S–H formed after hydration [28]. The weak absorption peak at 3455 cm^−1^ was caused by the O–H stretching vibration, which was a part of C–H and some unreacted hydrate particles [34]. The C–S–H and C–H decreased sharply in the images after the test. This indicated that these components were released into the solution through hydration and hydrolysis, converted into hydroxide after reacting with heavy metals and calcium reacting with sulphate.

**Figure 7:**
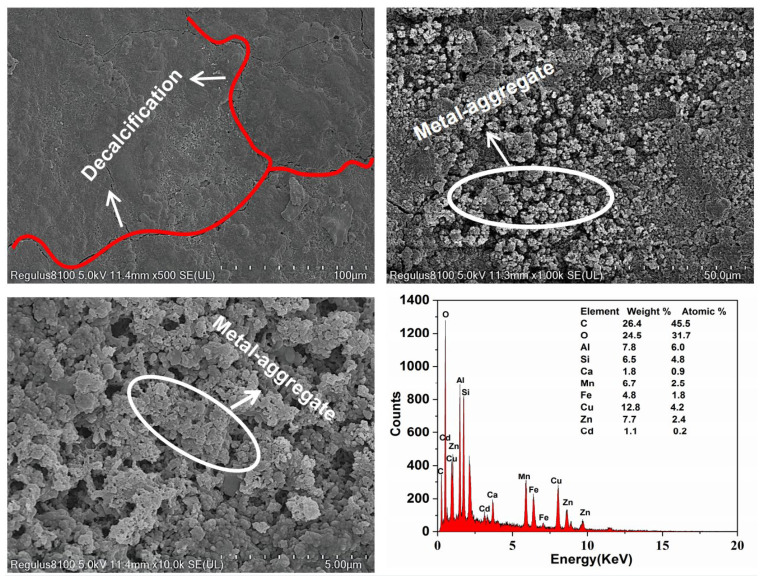
SEM patterns of RMPC after reaction.

Figure 7 shows the microstructural changes in the RMPC after the test. From Figure S3, there are no obvious cracks in the concrete before the experiment, which indicates good bonding between the concrete material. However, cracks appeared after the test (Figure 7). It was proven that RMPC was continuously eroded by wastewater during the process, including the alkaline cations released through silicate phase dissolution and by erosion of substances containing calcium by action of sulphate [35]. Dendritic particles appeared on the surface after the reaction (Figure 7). According to the increase in heavy metals (especially cadmium coming from nothing) shown in the composition detection results, it was inferred that there were clusters of heavy metal particles that were not yet formed during the process of adsorption [36].

#### 3.3.3. Mechanisms of Heavy Metal Removal by RMPC

Based on the above test results, it was inferred that the main mechanisms of heavy metal removal by RMPC were surface adsorption and chemical precipitation. C–S–H in RMPC was porous and loose and had a strong adsorption capacity, which adsorbed and solidified heavy metal ions on its surface. In addition, a certain amount of haematite and aluminite components in RMPC had relatively strong adsorption and solidification effects on the heavy metal ions. This was proven by the continuous formation of heavy–metal ion particle clusters on the concrete surface. The results also showed that the increase in heavy metals was limited; therefore, surface adsorption was not the main mechanism of heavy metal interception. Chemical precipitation was mainly caused by the reaction of OH^+^ and heavy metal ions in the solution to produce water–insoluble hydroxides, which was proved by the presence of large amounts of Mn(OH)_2_, Zn(OH)_2_, Cu(OH)_2_ and Cd(OH)_2_ in the sediment. Therefore, the removal of heavy metals was chiefly dependent on the amount of OH^+^ in the water.

**Figure 8:**
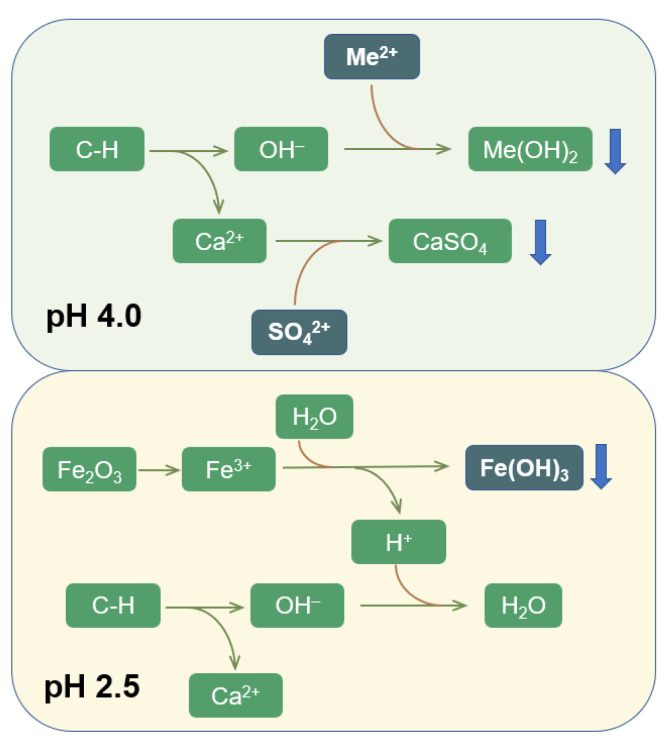
The mechanisms for removing heavy metals by RMPC from acid mine drainage (Me^2+^: Mn^2+^, Zn^2+^, Cu^2+^ and Cd^2+^).

The processes of wastewater entering the concrete are shown in Figure 8. The micro–melted C–H in the concrete underwent hydrolysis, releasing Ca^2+^ and OH^+^ into the water (C–H→Ca^2+^ + OH^−^), where the pH increased (Figure S1). When the input wastewater pH was 2.5, Fe(III), which originated from the haematite in the red mud, began to dissolve. The Fe(III) in the water hydrolysed preferentially (Fe^3+^ + 3H_2_O ⇋ Fe(OH)_3_ + 3H^+^ (pH < 3–3.7)) to form Fe(OH)_3_ and released large amounts of H^+^, which consumed OH^−^ released by the concrete. Consequently, the precipitation of other heavy metals decreased rapidly, and the removal efficiency of heavy metals decreased. When the input wastewater pH was ≥4, Fe (III) dissolution was reduced. The SO_4_^2−^ in the water began to react with Ca(II) and generated CaSO_4_ precipitate (pH > 3.5–4), which was found in the precipitate. This process greatly consumed the Ca(II) in water, which promoted C–H→Ca^2+^ + OH^−^ to tend towards the right. Thus, OH^−^ was constantly released, ensuring that there was sufficient OH^−^ to react with the heavy metal ions, which were constantly removed. 

Therefore, owing to its alkaline characteristics, RMPC has a strong chemical precipitation effect on heavy metals. Under the dual effects of the promotion of calcium sulphate precipitation and the inhibition of Fe(III) dissolution and hydrolysis, RMPC could continuously and completely remove heavy metals from weakly acidic (pH about ≥4) wastewater containing sulphate. 

### 3.4. Discussion on Applicability in Practical Engineering

Water samples were collected from drainage ditches in a mining area in Liuyang, Hunan Province, and then passed through the designed column test device for heavy metal treatment. The concentrations of heavy metals in the wastewater are shown in Tables S2 and S3. According to the requirements, the wastewater quality strictly complied with “Environmental Quality Standards for Surface Water (GB3838-2002)”. 

An economic analysis of RMPC was performed and compared with that of heavy metal–permeable interceptor materials. Red mud was used; therefore, the cost of cement was reduced by 50% during the preparation process. The use of RMPC significantly reduced the cost and provided a new resourceful utilisation of red mud, effectively reducing its impact on environmental pollution. Therefore, RMPC has better prospects with respect to economic and technical applications.

## 4. Conclusions

In this study, red mud was used to replace part of the cement to form a polymer and prepare pervious concrete, achieving its use as a resource. The removal efficiency of heavy metals of concrete with different red mud contents was studied through batch experiments. In addition, a laboratory pilot device was designed to test the treatment capacity of RMPC for acid mine drainage under different conditions, and the long–term treatment capacity and stability of RMPC under optimal conditions were studied. The internal mechanisms of the treatment and the limitations of applicability were studied. The following conclusions were drawn:

As the content of red mud in RMPC increased to 50%, the compressive strength of concrete decreased from 11.3 Mpa (without red mud) to 6.7 Mpa. Further increases in red mud content did not meet the compressive strength requirements. Interestingly, the removal efficiency of heavy metals from acid mine drainage reached a maximum when the red mud content in RMPC was 50%; reaching about 100%;

When the influent pH was 2.5, the efficiency of heavy metal removal decreased over time. However, when the influent pH was adjusted to 4.0, the removal efficiency of heavy metals in the system reached above 90%, and the system could achieve 32 weeks of long–term stable treatment. Therefore, when using PMPC to treat acidic mine drainage, the influent pH should be maintained at 4.0. In addition, there needs to be a sufficient area to provide the treatment system with hydraulic retention time of 20 h;

The main mechanism of heavy metal treatment by RMPC was the reaction of heavy metals with the OH^−^ produced by hydrolysis of CH, which produced hydroxide precipitation, accompanied by the adsorption and solidification of a small amount of C–S–H and haematite on the RMPC surface. When the wastewater pH was 2.5, the haematite in red mud dissolved and Fe(III) in the solution hydrolysed, inhibiting the precipitation of other heavy metals. The treatment capacity of RMPC was continuously reduced. At pH = 4, the dissolution of haematite decreased and CaSO_4_ began to precipitate. The large amount of SO_4_^2−^ in the solution consumed Ca(II) and continuously promoted the hydrolysis of CH. Thus, RMPC can achieve the long–term efficient removal of heavy metals;

RMPC could completely remove heavy metals from AMD samples obtained from the drainage ditch of a mining area in Liuyang, Hunan Province. The cost of RMPC was lower than that of conventional permeable wall materials, and the application of PMPC is expected to reduce the cost of permeable reactive barrier technologies in the treatment of acid mine drainage. The use of RMPC is also a novel way by which resource utilisation of red mud can be achieved. Therefore, RMPC has high practical value for heavy metal wastewater treatment.

## Figures and Tables

**Table 1:** Chemical compositions of red mud (RM) and Portland cement (OPC).

	SiO_2_	Al_2_O_3_	Fe_2_O_3_	CaO	MgO	Na_2_O	K_2_O	P_2_O_5_	TiO_2_	MnO
OPC	21	7.31	3.12	58.5	5.94	0.691	0.83	0.0926	0.378	0.156
RM	24.6	25.2	23.0	3.28	0.575	17.7	1.52	0.131	2.05	0.053

**Table 2:** Compositions of prepared pervious concretes.

Mix Design	Aggregate (kg/m^3^)	OPC (kg/m^3^)	RM (kg/m^3^)	Water (kg/m^3^)	Superplasticiser (kg/m^3^)
RM-0	1300	400	0	85.5	2
RM-25	1300	300	100	88	2
RM-50	1300	200	200	94.5	2

## Data Availability

Data are reported in the article and the Supplementary Materials.

## Supplementary Materials

The following supporting information can be downloaded at: https://www.mdpi.com/article/10.3390/polym14245355/s1. Table S1. Characteristics of simulated acid mine drainage. Table S2. Characteristics of real acid mine drainage from Qibao Shan. Table S3. Characteristics of effluent at different times. <LD indicates that value is below the detection limit. Figure S1. Variation of pH value in heavy metals removal experiment. Figure S2. Changes in Cd, Mn, Cu and Zn concentrations, and pH with time during long-term operation. Figure S3. SEM photo of raw RMPC before reaction.
