# Supplementary material for: Removal of Heavy Metals from Acid Mine Drainage by Red Mud–Based Geopolymer Pervious Concrete: Batch and Long–Term Column Studies
Source: Polymers (Basel). 2022 Dec 7;14(24):5355. doi: 10.3390/polym14245355 (PMC9787840; doi:10.3390/polym14245355)
Supplement: Supplementary file 1 [file polymers-14-05355-s001.zip › polymers-2053860-supplementary.pdf]

Supplementary data

1. Tables

Table S1. Characteristics of simulated acid mine drainage.

pH	Cu(mg/L)	Mn(mg/L)	Cd(mg/L)	Zn(mg/L)
4	10.0	10.0	1.6	16.0

Table S2. Characteristics of real acid mine drainage from Qibao Shan.

pH	Cd(mg/L)	Cu(mg/L)	Mn(mg/L)	Pb(mg/L)	Zn(mg/L)
4.8	1.0	35.2	3.1	0.3	3.2

Table S3. Characteristics of effluent at different times. <LD indicates that value is below the detection limit.

Days	Cd	Cu	Mn	Pb	Zn
1	<LD	0.01	0.01	<LD	<LD
5	<LD	0.03	0.01	<LD	<LD
10	<LD	0.02	0.02	<LD	<LD
15	<LD	0.02	0.03	<LD	<LD

3. Figures

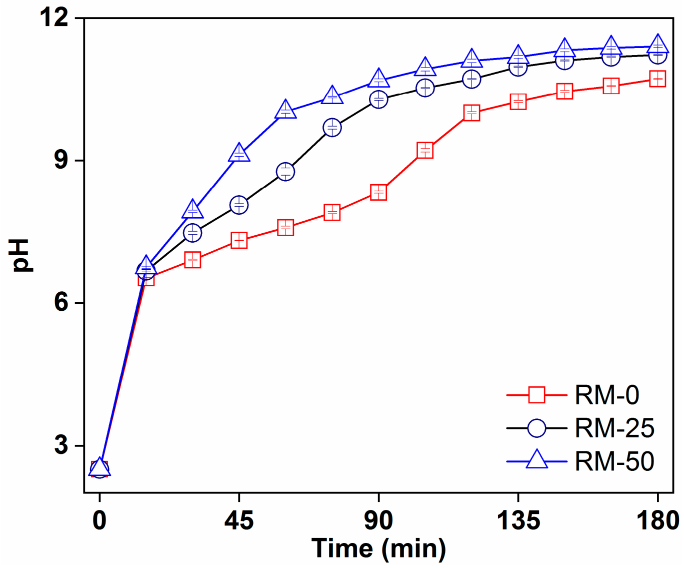

Figure S1. Variation of pH value in heavy metals removal experiment.

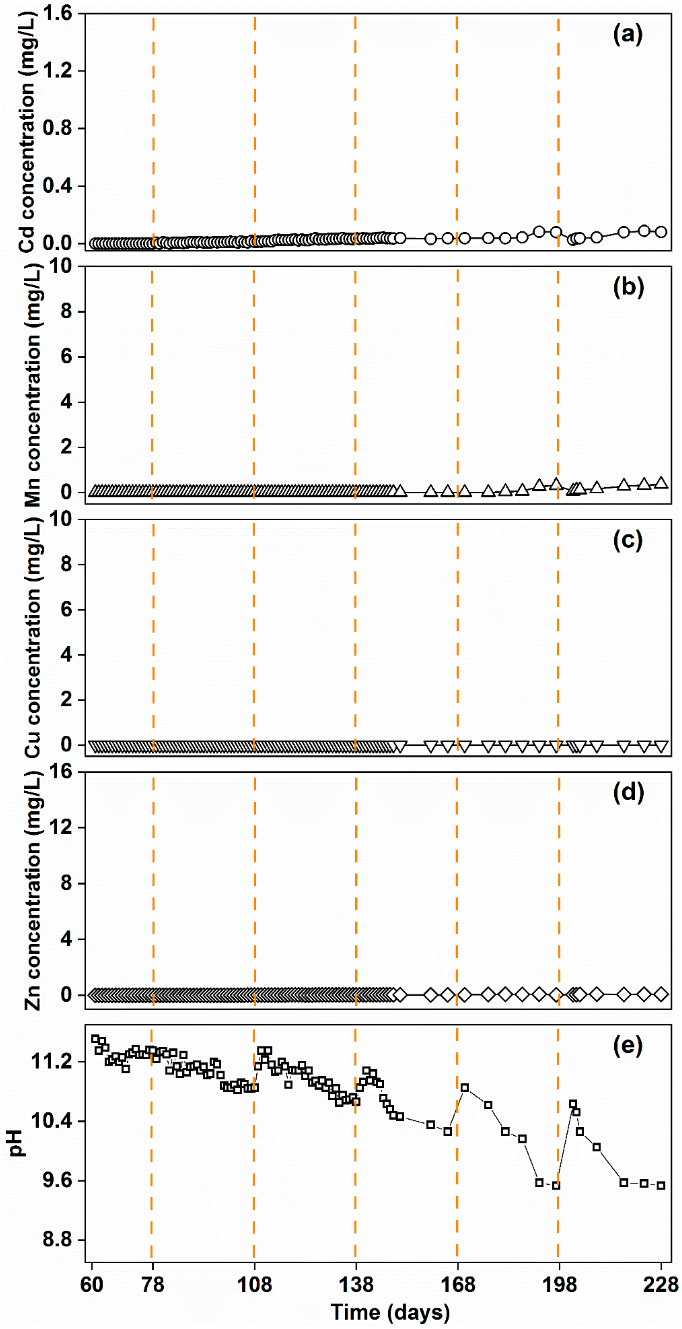

Figure S2. Changes in (a) Cd , (b) Mn, (c) Cu and (d) Zn concentrations, and (e) pH with time during long-term operation.

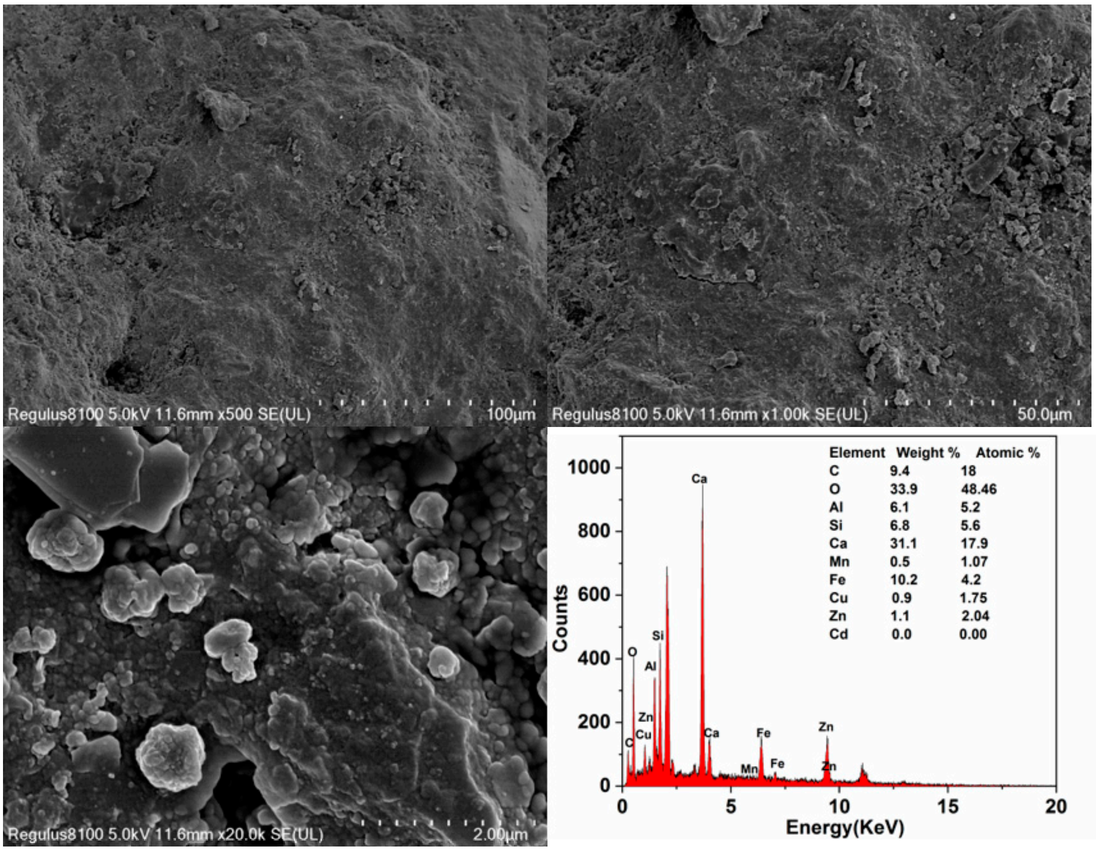

Figure S3. SEM photo of raw RMPC before reaction.
